# Functional Outcome and Safety of Intracranial Thrombectomy After Emergent Extracranial Stenting in Acute Ischemic Stroke Due to Tandem Occlusions

**DOI:** 10.3389/fneur.2018.00940

**Published:** 2018-11-20

**Authors:** Philipp Bücke, Marta Aguilar Pérez, Muhammad AlMatter, Victoria Hellstern, Hansjörg Bäzner, Hans Henkes

**Affiliations:** ^1^Neurologische Klinik, Klinikum Stuttgart, Stuttgart, Germany; ^2^Klinik für Neuroradiologie, Klinikum Stuttgart, Stuttgart, Germany

**Keywords:** tandem occlusions, acute ischaemic stroke (AIS), endovascular therapy, thrombectomy, extracranial stenosis, functional outcome, inhibition of platelet aggregation

## Abstract

**Background and Purpose:** Various endovascular approaches to treat acute ischemic stroke caused by extra- intracranial tandem occlusions (TO) exist: percutaneous transluminal angioplasty with or without emergent extracranial carotid stenting (ECS) due to high-grade stenosis preceded or followed by intracranial mechanical and/or aspiration thrombectomy (MT). Which treatment strategy to use is still a matter of debate.

**Methods:** From our ongoing prospective stroke registry we retrospectively analyzed 1,071 patients with anterior circulation stroke getting endovascular treatment within 6 h of symptom onset. ECS prior to intracranial MT for TO (*n* = 222) was compared to MT as standard of care (control group; acute intracranial vessel occlusion without concomitant ipsilateral ICA-occlusion or high-grade stenosis [C; *n* = 849]). Good functional outcome (mRS ≤ 2 at 3 months), mortality rates, frequencies of symptomatic intracranial hemorrhage (sICH) and successful recanalization (Thrombolysis in Cerebral Infarction Score [TICI] 2b or 3) were assessed. In subgroup analyses we tried to detect possible influences of stroke etiology, dual inhibition of platelet aggregation (IPA; clopidogrel [CLO]: *n* = 83; ticagrelor [TIC]: *n* = 137; in combination with Aspirin) and intravenous thrombolysis (IVT).

**Results:** Functional outcome was superior in TO (mRS 0–2: 44.6%) when compared with controls (36.0%; OR [95% CI]: 3.49 [1.59–7.67]; *p* = 0.002). There was no difference in all-cause mortality at 3 months (TO: 21.6%; C: 27.7%; 0.78 [0.47–1.29]; *p* = 0.324), in-hospital mortality (0.76 [0.45–1.30]; *p* = 0.324), sICH (TO: 3.2%; C: 5.0%; 0.70 [0.30–1.59]; *p* = 0.389), and TICI 2b/3 (TO: 89.1%; C: 88.3%; *p* = 0.813). In subgroup-analysis, TIC and CLO did not differ in functional outcome (TIC: 45.3%; CLO: 44.6%; 1.04 [0.51–2.09]; *p* = 0.920) and mortality rates (all-cause mortality: TIC: 23.4%; CLO: 16.9%; 0.75 [0.27–2.13]; *p* = 0.594). sICH was more frequent in TIC (*n* = 7 [5.1%]) vs. CLO (*n* = 0; *p* = 0.048).

**Conclusion:** In our pre-selected cohort, ECS prior to intracranial MT in TO allowed for a good functional outcome that was superior compared to a control population. Mortality rates did not differ. Despite a dual IPA in TO, there was no increase in sICH. CLO and TIC for dual IPA did not differ in terms out outcome and mortality rates. A significant increase in sICH was observed after initial loading with TIC.

## Introduction

Mechanical thrombectomy and/or aspiration thrombectomy (MT) in acute ischemic stroke due to embolic large vessel occlusion is effective and safe (1–5). Specific recommendations and national guidelines for indication, implementation, and patient selection exist (6). A considerable number of patients present with extracranial–intracranial tandem occlusions (TO; occlusion or high-grade stenosis of an extracranial internal carotid artery [ICA] with a concomitant ipsilateral intracranial large vessel occlusion) (7). Currently, there is a lack of guidance on how to treat those patients. Several endovascular treatment strategies are proposed: percutaneous transluminal angioplasty (PTA) with or without emergent stenting of the extracranial ICA (ECS) preceded or followed by MT (7–10). Which technique to use is still a matter of debate (11–14). We report data on TO where initial ECS is followed by MT (extracranial first).

## Materials and methods

### Study population

Consecutive patients from our prospective single-center stroke registry treated with MT between January 2010 and December 2017 were screened and retrospectively analyzed. Patients with an anterior circulation ischemic stroke caused by an occlusion of the ICA, the carotid-T, an M1- or M2-branch of the middle cerebral artery (MCA) were included. We did not consider distal MCA-occlusions, occlusions of the anterior cerebral artery or posterior circulation stroke. Proximal vessel occlusions (on initial imaging) that were found recanalized during angiography (spontaneously or as an effect of intravenous thrombolysis [IVT]) were removed from further analysis. Patients treated after 6 h of symptom onset or presenting with wake-up stroke or unknown symptom onset were excluded. We did not include cases of primary stent-angioplasty without MT (due to high-grade intra- or extracranial stenosis or dissection). Datasets without a 3-month follow-up as well as datasets including inconsistent information that could not be confirmed were excluded. Local institutional review board approval was obtained.

Patients either presented primarily in the emergency department of our neurovascular center or via hospitals within or surrounding the city of Stuttgart (secondary transfer) (15). Irrespectively, endovascular therapy was based upon the initial intention to treat patients (based on a shared decision-making concept including stroke-neurologists and interventional neuroradiologists) without further triage or additional imaging procedures prior to the intervention. General anesthesia was performed on a regular basis.

Information on baseline characteristics, current medication, symptom onset, stroke severity (e.g., National Institutes of Health Stroke Scale [NIHSS], modified Rankin Scale [mRS]) or periprocedural information (e.g., Thrombolysis in Cerebral Infarction Score [TICI]) were extracted from admission notes, internal documentation, referral, or discharge papers. Imaging modality and imaging times were stored in our Picture Archiving and Communication System. Follow-up information were collected by our study nurse (via telephone calls).

TO were defined as an occlusion or high-grade stenosis (NASCET [North American Symptomatic Carotid Endarterectomy Trial] >70%) of the extracranial ICA with a concomitant ipsilateral occlusion of the intracranial ICA, the carotid-T or the MCA (M1 or M2 branch). We used the extracranial first approach (emergent extracranial stenting followed by intracranial MT). The following patients defined the control group: (1) embolic intracranial large vessel occlusion; (2) absence of a high-grade intra- or extracranial stenosis requiring emergent stenting; (3) treatment within 6 h of symptom onset; (4) MT only without additional or primary stent application.

In subgroup analyses we tried to detect possible differences in TO due to etiology or medical treatment. (1) Baseline characteristics and outcome in extracranial atherosclerosis (LAD), dissection and in patients with competing etiologies (LAD/CE; both cardiac embolism [e.g., atrial fibrillation] and LAD being possible etiologies) were compared. All patients with atrial fibrillation were summarized in LAD/CE unless a definite cause of stroke (e.g., LAD) could be determined. (2) Loading. Prior to emergent stenting, aspirin (500 mg IV) in combination with either clopidogrel (CLO; 600 mg PO via a nasogastric tube) or ticagrelor (TIC; 180 mg PO via a nasogastric tube) was given to inhibit platelet aggregation. The choice of the respective drug was at the discretion of the interventional neuroradiologist.

Due to a faster inhibition in platelet aggregation (IPA), TIC is currently preferred if emergent stenting is required (16). To secure an immediate IPA (until the expected effect of TIC or CLO), we implemented a bridging concept with the glycoprotein IIb/IIIa inhibitor eptifibatide (given as a single body weight-adapted IV bolus) (17). Post intervention, there was strict blood pressure control (systolic blood pressure < 130 mm Hg) for a minimum of 3 days. Outcome and hemorrhagic complications in TIC and CLO were compared. After initial loading, dual IPA was continued for a minimum of 3 months (CLO [75 mg/day] or TIC [90 mg twice a day] in combination with aspirin 100 mg/day) followed by monotherapy with aspirin lifelong. Platelet function was assessed with Multiplate® and/or VerifyNow® tests. In case of incomplete IPA, CLO was replaced by TIC. Anticoagulation (in case of atrial fibrillation) was begun depending on stroke severity (NIHSS) and the size of the infarcted tissue (18). (3) Effects of IVT in both the control group and TO on outcome and hemorrhagic complications. (4) Potential factors influencing outcome and mortality in both controls and TO.

### Outcome measures

Good functional outcome (mRS 0-2 at day 90) was the primary outcome parameter. Secondary outcome measures were: (1) development of a symptomatic intracranial hemorrhage (sICH) according to the Solitaire™ with the Intention for Thrombectomy as Primary Endovascular Treatment for Acute Ischemic Stroke (SWIFT PRIME) criteria (parenchymal hemorrhage [PH] type 1 or 2, subarachnoid hemorrhage [SAH] or intraventricular hemorrhage within 24 h after MT with a deterioration in the NIHSS ≥4 points or leading to death) (19); (2) in-hospital mortality; (3) all-cause mortality (day 90).

### Statistical analysis

Numerical baseline characteristics were described in mean (standard deviation). Categorical baseline parameters were described in frequencies. Comparing groups, the Fisher's exact test (categorical parameters), the Kruskal-Wallis-test or the Mann-Whitney-*U*-test (numerical parameters) were used as appropriate. Analyzing more than two groups [e.g., subgroup analysis (1)] outcome in the respective group (e.g., LAD) was compared to the outcome in the remaining groups (e.g., LAD/CE and dissection). A multivariate logistic regression model (considering possible confounders [based on literature research; *p* < 0.05 in baseline characteristics]) tried to detect factors influencing outcome and mortality. A *p*-value below 0.05 was considered statistically significant. Stata/IC 13.1 for Windows (StataCorp LP, College Station, Texas, USA) was used for statistical analysis.

## Results

**Figure 1:**
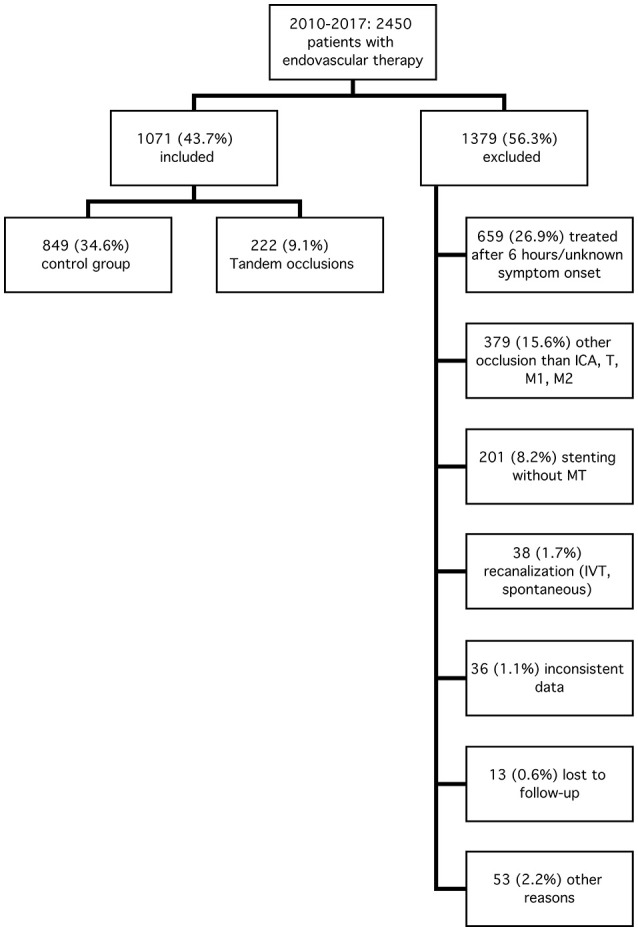
Inclusion and exclusion criteria. ICA, internal carotid artery; T, carotid-T; M1 (M2), M1 (M2), segment of the middle cerebral artery; MT, mechanical and/or aspiration thrombectomy; IVT, intravenous thrombolysis.

Between 2010 and 2017, *n* = 2450 acute ischemic stroke patients received endovascular recanalization therapy by us. *N* = 1071 (43.7%) met the predefined inclusion criteria, *n* = 1379 (56.3%) had to be excluded from further analysis (Figure 1). *N* = 222 patients were included in TO, *n* = 849 in the control group (C).

The baseline characteristics are shown in Table 1. There were significant differences (TO vs. C) in sex (male: 68.0 vs. 46.4%; *p* < 0.001), age (mean [SD]: 67.8 [12.8] vs. 73.1 [12.9]; *p* < 0.001), atrial fibrillation (27.5 vs. 62.5%; *p* < 0.001), smoking (25.3 vs. 8.6%; *p* < 0.001), IVT (23.9 vs. 31.7%; *p* = 0.026), baseline NIHSS (mean [SD]: 14.8 [8.1] vs. 16.5 [7.6]; *p* = 0.001), stroke etiology and target vessel (see Table 1). Time-to-recanalization was longer in TO (hours; mean [SD]: 6 (1.8) vs. 5.1 (1.5); *p* < 0.001) due to a longer duration of treatment (hours; TO: 2.3 (1.6); C: 1.5 (1.1); *p* < 0.001). There was no difference in onset-to-groin or imaging-to-groin times.

**Table 1:** Baseline characteristics, imaging findings, and time management.

	**Main analysis; baseline characteristics in tandem occlusions vs. controls**			**Subgroup analysis; baseline characteristics depending on etiology**		
	**TO**	**Controls**	***p*-value**	**Dissection**	**LAD**	**LAD/CE**	***p*-value**
	*n* = 222^*^	***n =* = 849**		***n =* 26**	***n =* 149**	***n =* 41**
**DEMOGRAPHICS**							
Age, mean (SD), years	67.8 (12.8)	73.1 (12.9)	< 0.001	48.1 (10.7)	69.2 (10.3)	75.4 (10.4)	< 0.001
Male sex, *n* (%)	151 (68.0)	394 (46.4)	< 0.001	19 (73.1)	105 (70.5)	23 (56.1)	0.194
**CARDIOVASCULAR RISK PROFILE**, ***n*** **(%)**							
Atrial fibrillation	61 (27.5)	531 (62.5)	< 0.001	0 (n.a.)	22 (14.8)	39 (95.1)	< 0.001
Hypertension	165 (74.3)	583 (68.7)	0.118	12 (46.2)	119 (79.9)	32 (78.0)	0.002
Diabetes mellitus II	48 (21.6)	180 (21.2)	0.927	2 (7.7)	38 (25.5)	8 (19.5)	0.114
Hyperlipidemia	51 (23.0)	179 (21.1)	0.582	2 (7.7)	37 (24.8)	12 (29.3)	0.093
Smoking	52 (23.4)	73 (8.6)	< 0.001	7 (26.9)	39 (26.2)	6 (14.6)	0.307
Coronary artery disease	48 (21.6)	231 (27.2)	0.103	2 (7.7)	27 (18.2)	19 (47.3)	< 0.001
**STROKE ETIOLOGY**, ***n*** **(%)**							
Atherothrombotic (LAD)	149 (67.1)	23 (2.7)	< 0.001	n.a.	n.a.	n.a.	n.a.
Cardioembolic	0 (n.a.)	537 (63.3)	< 0.001	n.a.	n.a.	n.a.	n.a.
Dissection	26 (11.7)	1 (0.1)	< 0.001	n.a.	n.a.	n.a.	n.a.
ESUS ^‡^	0 (n.a.)	263 (31.0)	< 0.001	n.a.	n.a.	n.a.	n.a.
LAD/CE (LAD + cardioembolic)	41 (18.5)	0 (n.a.)	< 0.001	n.a.	n.a.	n.a.	n.a.
Other etiology	6 (2.7)	25 (2.9)	1.000	n.a.	n.a.	n.a.	n.a.
**NIHSS SCORE (BASELINE)**							
Mean (SD)	14.8 (8.1)	16.5 (7.6)	< 0.001	14.7 (8.2)	14.6 (8.5)	15.9 (7.0)	0.495
**IMAGING MODALITY**, ***n*** **(%)**							
MRI	52 (23.7)	177 (21.2)	0.462	9 (34.2)	35 (23.8)	6 (15.0)	0.204
**MOST PROXIMAL VESSEL OCCLUSION**, ***n*** **(%)**							
ICA	14 (6.3)	22 (2.6)	0.030	3 (11.5)	6 (4.0)	3 (7.3)	0.093
Carotid-T	58 (26.1)	195 (23.0)		7 (26.9)	38 (25.5)	11 (26.8)
MCA, M1	123 (55.4)	533 (62.8)		9 (34.6)	90 (60.4)	22 (53.7)
MCA, M2	27 (12.2)	99 (11.7)		7 (26.9)	15 (10.1)	5 (12.2)
IVT, *n* (%)	53 (23.9)	269 (31.7)	0.026	7 (26.9)	39 (26.2)	5 (12.2)	0.147
**TIME MANAGEMENT (HOURS), MEAN (SD)**							
Time to recanalization	6.0 (1.8)	5.1 (1.5)	< 0.001	7.4 (2.3)	5.6 (1.6)	6.2 (1.9)	< 0.001
Symptom-onset to groin puncture	3.6 (1.1)	3.7 (1.9)	0.832	4.0 (1.3)	3.5 (1.0)	3.8 (1.1)	0.135
Imaging to groin puncture	2.3 (0.8)	2.3 (0.8)	0.531	2.3 (1.1)	2.2 (0.8)	2.4 (0.8)	0.343
Duration of treatment	2.3 (1.6)	1.1 (1.2)	< 0.001	3.4 (2.6)	2.1 (1.3)	2.5 (1.5)	< 0.001
**THROMBECTOMY DEVICES**, ***n*** **(%)**							
Penumbra ACE aspiration	7 (3.2)	28 (3.3)	0.220	1 (3.8)	5 (3.4)	1 (2.4)	0.142
Solitaire stent retriever	13 (5.9)	33 (3.9)		2 (7.7)	5 (3.4)	6 (14.6)
Phenox stent retriever	156 (70.3)	626 (73.7)		19 (73.1)	108 (72.5)	26 (63.4)
MicroVention aspiration	33 (14.9)	105 (12.4)		2 (7.7)	25 (16.8)	4 (9.8)
Other	13 (5.9)	57 (6.8)		2 (7.7)	6 (4.1)	4 (9.8)
**HEMORRHAGIC COMPLICATIONS**, ***n*** **(%)**							
PH1	7 (3.2)	32 (3.8)	0.841	1 (3.8)	5 (3.4)	1 (2.4)	1.000
PH2	9 (4.1)	33 (3.9)	0.848	0 (n.a.)	8 (5.4)	1 (2.4)	0.655
SAH	29 (13.1)	60 (7.1)	0.006	6 (23.1)	20 (13.4)	2 (4.9)	0.091
Successful recanalization (TICI 2b/3), *n* (%)	197 (89.1)	748 (88.3)	0.813	23 (88.5)	134 (89.9)	35 (87.5)	0.834

*TO, tandem occlusion; LAD, large artery disease/atherothrombotic; LAD/CE, competing etiologies (LAD plus cardiac embolism); SD, standard deviation; IVT, intravenous thrombolysis; PH, parenchymal hemorrhage; SAH, subarachnoid hemorrhage; n.a., not applicable; TICI, Thrombolysis in Cerebral Infarction; NIHSS, National Institutes of Health Stroke Scale*.

* *n, total number of patients included in the respective group; might differ from n in any category of baseline characteristics due to missing data*.

‡ *ESUS, embolic stroke of undetermined source*.

Good functional outcome was more frequent in TO (44.6%) compared to controls (36.0%; OR [95% CI] 3.49 [1.59–7.67]; *p* = 0.002; adjusted for differences in baseline characteristics; Table 2). We did not observe differences in in-hospital mortality (0.76 [0.45–1.30]; *p* = 0.324), all-cause mortality after 3 months (0.78 [0.47–1.29]; *p* = 0.324) and the frequency of sICH (0.70 [0.30–1.59]; *p* = 0.389). Postinterventional SAH was more common in TO (13.1%) than in controls (7.1%; *p* = 0.006).

**Table 2:** Outcome and safety parameters.

	**TO**	**Controls**	**OR (95% CI)**	***p*-value**
mRS 0-2 (vs. mRS 3–6) at 3 months, *n* (%)	99 (44.6)	306 (36.9)	3.49 (1.59–7.67)	0.002
In-hospital mortality (mRS 6 vs. 0–5), *n* (%)	35 (15.8)	161 (19.0)	0.76 (0.45–1.30)	0.324
All-cause mortality at 3 months (mRS 6 vs. 0–5), *n* (%)	48 (21.6)	235 (27.7)	0.78 (0.47–1.29)	0.324
sICH (yes vs. no), *n* (%)	7 (3.2)	41 (5.0)	0.70 (0.30–1.59)	0.389

*TO, tandem occlusion; OR, Odds ratio; CI, confidence interval, mRS; modified Rankin Scale; sICH, symptomatic intracranial hemorrhage*.

The difference in baseline characteristics depending on etiology (LAD [*n* = 149], LAD/CE [*n* = 41], dissection [*n* = 26]) are shown in Table 1 (six patients were diagnosed with other etiologies than the above-mentioned and were not included in this analysis). Good functional outcome was inferior in LAD/CE (24.4%; 0.34 [0.16–0.75]; *p* = 0.005) compared to LAD (47.7%; 1.63 [0.90–2.97]; *p* = 0.138) and dissection (53.8%; 1.57 [0.69–3.59]; *p* = 0.299; Table 3). In-hospital mortality was higher in LAD/CE (29.3%; 3.03 [1.32–6.95]; *p* = 0.014) compared to LAD (12.8%; 0.55 [0.26–1.19]; *p* = 0.152) and dissection (7.7%; 0.43 [0.10–1.92]; *p* = 0.299). All-cause mortality was 15.4% in patients with dissection (0.64 [0.21–1.97; *p* = 0.610) vs. 17.4% in LAD (0.50 [0.25–0.98; *p* = 0.048) and 39.0% in LAD/CE (3.09 [1.45–6.61; *p* = 0.005). There was no significant difference in sICH (dissection: 0%; LAD: 4.1%; LAD/CE: 2.4%).

**Table 3:** Subgroup analysis.

	**mRS 0–2 (vs. 3-6) at 3 months**		**In-Hospital Mortality**		**All-Cause Mortality (3 months)**		**sICH**	
	**mRS 0–2, *n* (%)**	OR (95% CI); p^*^	**mRS 6, *n* (%)**	**OR (95% CI); *p***	**mRS 6, *n* (%)**	**OR (95% CI); *p***	**sICH yes, *n* (%)**	**OR (95% CI); *p***
**A: Etiology (Dissection, LAD, COMP)**								
Dissection	14 (53.8)	1.57 (0.69–3.59); 0.299	2 (7.7)	0.43 (0.10–1.92); 0.384	4 (15.4)	0.64 (0.21–1.97); 0.610	0 (n.a.)	n.a.; 1.000
LAD	71 (47.7)	1.63 (0.90–2.97); 0.138	19 (12.8)	0.55 (0.26–1.19); 0.152	26 (17.4)	0.50 (0.25–0.98); 0.048	6 (4.1)	2.79 (0.33–23.87); 0.439
LAD/CE	10 (24.4)	0.34 (0.16–0.75); 0.005	12 (29.3)	3.03 (1.32–6.95); 0.014	16 (39.0)	3.09 (1.45–6.61); 0.005	1 (2.4)	0.70 (0.08–6.01); 1.000
	**mRS 0**–**2 (vs. 3–6) at 3 months**		**In-Hospital Mortality**		**All-Cause Mortality (3 months)**		**sICH**	
	**mRS 0–2**, ***n*** **(%)**	**OR (95% CI);** ***p***	**mRS 6**, ***n*** **(%)**	**OR (95% CI);** ***p***	**mRS 6**, ***n*** **(%)**	**OR (95% CI);** ***p***	**sICH yes, n (%)**	**OR (95% CI);** ***p***
**B: Loading (ticagrelor/aspirin vs. clopidogrel/aspirin)**								
Clopidogrel	37 (44.6)	1.04 (0.51–2.09); 0.920	9 (10.8)	0.46 (0.17–1.25); 0.129	14 (16.9)	0.75 (0.27–2.13); 0.594	0 (n.a.)	n.a.; 0.048
Ticagrelor	62 (45.3)		24 (17.5)		32 (23.4)		7 (5.1)

*LAD, large artery disease; LAD/CE, competing etiologies (LAD and cardiac embolism); mRS, modified Rankin Scale; OR, Odds ratio; CI, confidence interval; sICH, symptomatic intracranial hemorrhage; n.a., not applicable*.

* *p-value for group comparison; outcome in one subgroup (e.g., dissection) vs. the remaining subgroups (LAD and LAD/CE)*.

In TO, *n* = 83 patients received a pre-medication with aspirin and CLO, *n* = 137 with aspirin and TIC. Two patients had to be removed from analysis because of inconsistent information. There was no difference in good functional outcome (adjusted for differences in respective baseline characteristics; CLO vs. TIC: 44.6 vs. 45.3%; 1.04 [0.51–2.09]; *p* = 0.920), in-hospital mortality (10.8 vs. 17.5%; 0.46 [0.17–1.25]; *p* = 0.129) and all-cause mortality after 90 days (16.9 vs. 23.4%; 0.75 [0.27–2.13]; *p* = 0.594; Table 3). sICH were more frequent in TIC (*n* = 7; 5.1%) compared to CLO (*n* = 0; *p* = 0.048; due to the total *n* = 7, a multivariate logistic regression for sICH was not calculated) as were SAH (CLO: 4,8%; TIC: 18.2%; *p* = 0.004). PH1 (CLO: 0%; TIC: 4.4%; *p* = 0.086) and PH2 (CLO: 1.2%; TIC: 5.8%; *p* = 0.158) were more frequent in CLO without reaching statistical significance. TICI 2b/3 was observed in 95.2% (CLO) and 86.0% (TIC) of patients respectively (*p* = 0.040).

In univariate analysis in controls, good functional outcome was observed in 40.9% of patients treated with IVT prior to endovascular therapy vs. 33.8% in non-IVT patients (1.36 [1.01–1.83]; *p* = 0.046). All-cause mortality after IVT (in controls) was 18.6 vs. 31.9% (0.49 [0.34–0.70]; *p* < 0.001). In TO, there was no such difference (IVT vs. non-IVT; mRS 0–2: 43.4 vs. 45.0%; 0.94 [0.50–1.75]; *p* = 0.875; mRS 6 at 3 months: 20.8 vs. 21.9%; 0.93 [0.44–2.00]; *p* = 1.000). IVT had no influence on the development of sICH in both controls (IVT vs. non-IVT: 5.7 vs. 4.6%; 1.25 [0.65–2.41]; *p* = 0.495) and TO (1.9 vs. 3.6%; 0.53 [0.06–4.53]; *p* = 1.000). In our cohort, there was no clear association between IVT and TICI 2b/3 in TO (IVT vs. non-IVT: 88.7 vs. 89.3%; 0.94 [0.35–2.51]; *p* = 1.000) or controls (89.9 vs. 87.6%; 1.26 [0.79–2.01]; *p* = 0.359). In multivariate analysis, IVT was (in controls only) associated with a reduction in all-cause mortality (0.46 [0.30–0.69]; *p* < 0.001, Table 4).

**Table 4:** Multivariate logistic regression (good functional outcome, all-cause mortality).

	**mRS 0–2 (vs. 3–6) at 3 months**		**mRS 6 (vs. 0–5) at 3 months**	
	**OR (95% CI)**	***p*-value**	**OR (95% CI)**	***p*-value**
**A: Control group**				
Male (vs. female)	n.s.	n.s.	n.s.	n.s.
Atrial fibrillation	n.s.	n.s.	0.50 (0.34–0.76)	0.001
Diabetes mellitus type II	n.s.	n.s.	n.s.	n.s.
Hyperlipidemia	n.s.	n.s.	n.s.	n.s.
Smoker	n.s.	n.s.	n.s.	n.s.
Hypertension	n.s.	n.s.	0.53 (0.36–0.80)	0.002
Coronary artery disease	n.s.	n.s.	n.s.	n.s.
TICI 2b/3	2.30 (1.09–4.90)	0.030	0.43 (0.25–0.74)	0.002
Vessel occlusion: M2	n.s.	n.s.	n.s.	n.s.
IVT	n.s.	n.s.	0.43 (0.28–0.66)	< 0.001
Age	0.94 (0.93–0.96)	< 0.001	1.09 (1.06–1.11)	< 0.001
NIHSS	0.90 (0.87–0.92)	< 0.001	1.10 (1.07–1.13)	< 0.001
Duration of treatment	0.53 (0.42–0.66)	< 0.001	1.20 (1.00–1.43)	0.050
Symptom-onset to groin puncture	0.72 (0.60–0.85)	< 0.001	1.50 (1.24–1.81)	< 0.001
Time to recanalization	0.72 (0.60–0.85)	< 0.001	1.33 (1.17–1.52)	< 0.001
**B: Tandem occlusions**				
Male (vs. female)	n.s.	n.s.	n.s.	n.s.
Atrial fibrillation	0.34 (0.14–0.81)	0.015	n.s.	n.s.
Diabetes mellitus type II	0.31 (0.13–0.73)	0.007	n.s.	n.s.
Hyperlipidemia	2.99 (1.17–7.60)	0.022	0.10 (0.03–0.38)	0.001
Smoker	n.s.	n.s.	n.s.	n.s.
Hypertension	n.s.	n.s.	n.s.	n.s.
Coronary artery disease	n.s.	n.s.	2.43 (1.00–5.90)	0.049
TICI 2b/3	^*^	^*^	n.s.	n.s.
Vessel occlusion: M2	3.54 (1.07–11.67)	0.038	n.s.	n.s.
IVT	n.s.	n.s.	n.s.	n.s.
Age	0.95 (0.92–0.99)	0.007	1.10 (1.05–1.16)	< 0.001
NIHSS	0.90 (0.86–0.95)	< 0.001	1.06 (1.01–1.11)	0.31
Duration of treatment	0.54 (0.35–0.82)	0.004	1.58 (1.23–2.03)	< 0.001
Symptom-onset to groin puncture	n.s.	n.s.	n.s.	n.s.
Time to recanalization	n.s.	n.s.	1.57 (1.26–1.95)	< 0.001

*mRS, modified Rankin Scale; OR, Odds ratio; CI, confidence interval; NIHSS, National Institutes of Health Stroke Scale; TICI, Thrombolysis In Cerebral Infarction; n.s., not significant; M2, M2-Segment of the middle cerebral artery; IVT; intravenous thrombolysis*.

* *all patients with TICI 0-2a presented with an mRS 3–6 at follow-up; TICI 0-2 is a perfect predictor for a negative outcome in this subgroup (TO)*.

To detect factors influencing outcome, separate multivariate logistic regression models were calculated for TO and controls (Table 4). Age (mRS 0–2: TO: 0.95 [0.92–0.99], *p* = 0.007; C: 0.94 [0.93–0.96], *p* < 0.001; mRS 6: TO: 1.10 [1.05–1.16], *p* < 0.001; C: 1.09 [1.06–1.11], *p* < 0.001) and NIHSS (mRS 0-2; TO: 0.90 [0.86–0.95], *p* < 0.001; C: 0.90 [0.87–0.92], *p* < 0.001; mRS 6: TO: 1.06 [1.01–1.11], *p* = 0.031; C: 1.10 [1.07–1.13], *p* < 0.001) showed significant associations. In TO, there was a negative association with atrial fibrillation (0.34 [0.14–0.81], *p* = 0.015) and diabetes mellitus type II (0.31 [0.13–0.73], *p* = 0.007) while hyperlipidemia (2.99 [1.17–7.60], *p* = 0.022) and target vessel M2 (3.54 [1.07–11.67], *p* = 0.038) were shown to be associated with a good outcome (2.99 [1.17–7.60], *p* = 0.022). In controls, TICI 2b/3 was significantly correlated with good functional outcome (2.30 [1.09–4.90], *p* = 0.030) and all-cause mortality (0.43 [0.25–0.74, p = 0.002). In TO, TICI 2b/3 could not be considered in multivariate analysis (for good functional outcome) as all patients with TICI 0-2a had mRS 3–6 in 3 month follow-up. In this cohort, TICI 0-2a was a perfect negative predictor. Time dependency in controls was evident for both symptom-onset to groin time (0.72 [0.60–0.85], *p* < 0.001) and duration of treatment (0.53 [0.42–0.66], *p* < 0.001). In TO, only duration of treatment (0.54 [0.35–0.82], *p* = 0.004) but not symptom-to-groin time or time to recanalization correlated significantly with outcome. Factors associated with mortality are shown in Table 4.

## Discussion

The main finding of our study was that outcome after endovascular therapy in TO using the extracranial first approach was shown to be superior compared to controls (patients with an acute ischemic stroke due to intracranial large vessel occlusion without concomitant high-grade extracranial stenosis of the ipsilateral ICA). Mortality rates did not differ. Despite the need of dual IPA after emergent stenting in TO, we did not observe an increase in sICH when compared to controls.

Evidence on endovascular treatment strategies in TO is mixed and inconsistent (7, 8, 10). Some authors suggest intracranial MT to be done before ECS (intracranial first) in order to minimize the time of critical hypoperfusion (8, 14). Currently, this seems to be the most widespread approach (9). However, ECS requires dual IPA which–especially in acute ischemic stroke–might increase the rate of hemorrhagic complications. Therefore, it is argued that a PTA should be performed in the acute setting while ECS in general (or vascular surgery) has to follow later during the course of the disease (9). Besides in an increase in (asymptomatic) SAH we did not observe additional hemorrhagic complications in our cohort. Most importantly, dual IPA did not increase the frequency of sICH. A third proposed treatment option is extracranial first (7, 9, 10). Immediately recanalizing the proximal ICA might improve or uphold crucial collateralization (21, 22). Asymptomatic ICA-stenosis can be compensated through a change in cerebral blood flow via existing or established collaterals (e.g., the Circle of Willis). A consistent reduction in cerebral perfusion (due to ICA-stenosis) might induce additional collaterals. Atherothrombotic stroke therefore is said to have greater collateral recruitment compared to stroke due to other etiologies (23). Together with possible effects of ischemic preconditioning of the brain, this could have a positive effect on outcome in TO (24). Yet, in progressive ICA-stenosis or acute occlusion, those compensation mechanisms might fail (25–29). In our cohort, time to recanalization was significantly longer in TO. Unlike the control population, where an association between symptom-onset to groin puncture time and good functional outcome was shown, symptom-onset to groin puncture time (and time to recanalization) did not significantly influence outcome in TO. This might also emphasize the importance of pre-existing collaterals. So far, superiority of any endovascular approach in TO could not be demonstrated (10). An average symptom-onset to groin puncture time of 3.6 h (TO; 3.7 h in controls) is attributed to the high number of secondary referrals (15).

The use of dual IPA in TO did not lead to an increase in symptomatic intracranial hemorrhages. The frequency of asymptomatic SAH (as seen on follow-up imaging) was significantly higher in TO. CLO is widely used in dual IPA after coronary, extra- or intracranial stenting. After loading, it takes 6 to 12 h until a sufficient inhibition in platelet activity is established (16). IPA in TIC is established after about 3 h (16). A faster IPA might reduce early complications (e.g., stent thrombosis) and therefore add an extra benefit. However, especially in dual IPA, TIC might introduce additional hemorrhagic complications (30, 31). From 2016/2017 onwards, we loaded patients with TIC and aspirin on a regular basis. Comparing CLO and TIC, we did not observe differences in outcome. Similar to previous data, there was a significant increase in major and minor hemorrhagic complications (sICH, SAH) after pre-medication with TIC (30, 31).

In our dataset, outcome after cervical ICA-dissection and LAD was superior compared to patients with LAD/CE (both LAD and cardioembolic etiology possible). Mortality in dissection was significantly lower. Favorable outcome in dissections–patients are younger without a typical cardiovascular risk profile—is well-known (32). In contrast, patients with cardioembolic stroke due to atrial fibrillation are older, stroke is more severe and has a higher risk of recurrence (33). Outcome in cardioembolic stroke, even after MT in a reasonable time window—is inferior to stroke caused by other etiologies (34, 35). The combination of LAD (or vascular disease in general) and atrial fibrillation has a further negative effect on functional outcome (36, 37). Other potential factors such as contraindications (e.g., major stroke and necessity of anticoagulation) or triple therapy are open for discussion.

Age, NIHSS and successful reperfusion have been identified as prognostic parameters in TO before (20). In our cohort, age and NIHSS showed a similar correlation in both TO and controls. TICI 2b/3 was associated with good functional outcome and a reduction in all-cause mortality in controls. In TO, TICI 0-2a was a perfect predictor for a poor outcome as all patients with TICI 0-2a got mRS 3–6 after 3 months. IVT is said to be associated with successful reperfusion in patients with TO (38, 39). In univariate analysis, we observed better functional outcome and reduced all-cause mortality in controls treated with IVT prior to MT (but not in TO). In TO, IVT was safe and did not lead to an increase in sICH. As shown before, we observed a beneficial effect of high cholesterol levels (in TO) on short-term outcome (40).

The main limitation of the study is its retrospective design. There is no information on patients where MT was not considered which might introduce selection bias. A certain inconsistency in decision-making has to be expected. Small sample sizes in subgroup analyses might lead to a power problem resulting in a potential underestimation of therapeutic or predictive effects. As there was no common study protocol, there is no information on potential contraindications or off-label decisions (e.g., IVT, dual IPA, individual healing attempts).

## Conclusion

Endovascular therapy in acute ischemic stroke due to TO using the extracranial first approach allows for a good functional outcome. In our cohort, dual IPA after emergent stenting in acute ischemic stroke patients was safe and did not lead to an overall increase in the frequency of sICH. Sufficiently powered randomized-controlled trials are needed for direct comparison of the different therapeutic strategies. In subgroup analysis focusing on dual IPA, TIC, and CLO did not differ in terms of outcome and mortality. However, TIC lead to a significant increase in sICH and SAH when compared to CLO. IVT-admission did not cause additional hemorrhagic complications.

## Data availability statement

All relevant data is contained within the manuscript.

## Author contributions

PB: study concept and drafting of the manuscript. All authors contributed to the acquisition, analysis and interpreting of data, and to the critical revision and final approval of the manuscript.

### Conflict of interest statement

The authors do not refer to specific medical devices since they are beyond the scope of this work. HH is co-founder and shareholder of phenox GmbH. MA has a proctoring and consulting agreement with phenox GmbH. The remaining authors declare that the research was conducted in the absence of any commercial or financial relationships that could be construed as a potential conflict of interest

## Abbreviations

CI: confidence interval
CLO: clopidogrel
LAD: large artery disease
LAD/CE: competing etiologies (LAD and cardiac embolism)
ECS: emergent extracranial carotid stenting
ESUS: embolic stroke of undetermined source
ICA: internal carotid artery
IPA: inhibition of platelet aggregation
IVT: intravenous thrombolysis
mRS: modified Rankin Scale
MCA: middle cerebral artery
MT: mechanical and/or aspiration thrombectomy
NIHSS: National Institutes of Health Stroke Scale
PH: parenchymal hemorrhage
PTA: percutaneous transluminal angioplasty
SAH: subarachnoid hemorrhage
SD: standard deviation
sICH: symptomatic intracerebral hemorrhage
TIC: ticagrelor
TICI: Thrombolysis in Cerebral Infarction Score
TO: tandem occlusion.
